# Breast Cancer Patients’ Perceptions of Their Experience With Chemotherapy-Induced Nausea and Vomiting and Its Impact on Quality of Life in Jeddah, Saudi Arabia

**DOI:** 10.7759/cureus.12038

**Published:** 2020-12-12

**Authors:** Ahmed B Ilyas, Reem K Bahaj, Azzam A Shaikh, Bashaer S Khawandanah, Meteb Al-Foheidi, Tagwa Y Omer

**Affiliations:** 1 Oncology, Umm Al-Qura University, Mecca, SAU; 2 Oncology, King Abdulaziz University Faculty of Medicine, Jeddah, SAU; 3 Medical Oncology, College of Medicine, King Saud Bin Abdulaziz University for Health Sciences, Jeddah, SAU; 4 Nursing, College of Nursing, King Saud Bin Abdulaziz University for Health Sciences, Jeddah, SAU

**Keywords:** breast neoplasms, quality of life, antiemetics, surveys and questionnaires, antineoplastic agents, nausea, vomiting, saudi arabia, female, chemotherapy induced nausea and vomiting (cinv)

## Abstract

Background

Breast cancer accounts for 11.6% of all neoplasms worldwide and is the commonest cancer among Saudi females (29.7%). Chemotherapy-induced nausea and vomiting (CINV) is a very common side effect of chemotherapy that has a great impact on the quality of life (QoL) of patients. Literature is still scarce about this effect on the Saudi population. This study aims to explore breast cancer patients’ perception of their experience with CINV and its impact on QoL.

Methods

This is a cross-sectional retrospective study conducted on Saudi adult female breast cancer patients who underwent chemotherapy at King Abdulaziz Medical City, Jeddah, Saudi Arabia. Data collected through patients’ records review, face-to-face and telephone structured interviews using a questionnaire composed of four parts: sociodemographic characteristics, nature of acute CINV (within 24 hours) and delayed CINV (after 24 hours), impact on QoL, and general information on their experience.

Results

Out of a total population of 173, 98 (56.65%) patients participated in the study. The main findings show that 78.6% experienced nausea, whereas 35.7% experienced vomiting. Most participants had a moderate-to-extreme impact on their QoL due to nausea (74.0%) and vomiting (62.9%). Overall, 57.5% rated anti-emetics as excellent for controlling CINV, whereas 22.9% rated them as moderate to good; 83.5% were completely compliant on anti-emetics and 71.1% reported that they received completely comprehensive education about CINV. Religious practices (74.4%), diet (57.7%), and relaxation techniques (44.9%) were found to be the most common non-pharmacological methods used to control CINV. No significant correlation was found between the effect of CINV on QoL and sociodemographic characteristics (p > 0.05).

Conclusions

CINV is very common among Saudi adult female breast cancer patients; despite being completely compliant and receiving comprehensive education and effective anti-emetics; CINV still had a high impact on different aspects of QoL. Health care professionals should consider CINV as an issue and should find effective strategies for alleviating patients’ suffering.

## Introduction

According to the 2018 World Health Organization global cancer project, breast cancer is the fifth leading cause of cancer death worldwide and is considered - along with lung cancer - the most common cancer worldwide. It accounts for 11.6% of all neoplasms in the world, with 2,088,849 new cases diagnosed annually, and a cause of 626,679 deaths in 2018 [1]. In Saudi Arabia specifically, breast cancer is considered the second leading cause of cancer death and is the most common cancer among Saudi females representing 29.7% of all cancers [2].

Breast cancer patients are treated commonly with a combination of anthracycline and cyclophosphamide, which are classified as high emetogenic chemotherapy (HEC) according to several organizations’ guidelines such as the American Society of Clinical Oncology (ASCO), National Comprehensive Cancer Network (NCCN), the European Society for Medical Oncology (ESMO), and the Multinational Association of Supportive Care in Cancer (MASCC), necessitating administration of the anti-emetics [3-5]. In addition, other non-pharmacological treatments with varying efficacies were used by patients for treating CINV, such as acupressure, exercise, diet, hypnosis, cognitive distraction, and praying [6-8].

Despite the latest improvements in anti-emetics drugs administration, CINV is still considered one of the worst side effects of chemotherapy, impairing the patient’s quality of life (QoL), and if left untreated, it can affect more than 90% of patients on HEC, consequently affecting their willingness to continue chemotherapy [9]. A Portuguese study showed that 77% of female breast cancer patients reported nausea and 50% reported vomiting at least once [10]. In Jordan, a research that surveyed cancer patients showed that most had nausea (71.4%) and vomiting (57.3%) [11]. Based on the severity of the CINV, the patient may even demand reducing or modifying the chemotherapy dose, negatively influencing the achievement of therapy and patients' general survival [12].

CINV can be broadly classified into three categories: anticipatory (a learned experience that may lead to pre-chemotherapy CINV), acute (occurs in the first 24 hours post-chemotherapy), and delayed (occurs after 24 hours of chemotherapy) [3]. Factors that affect CINV can be divided into two classifications: treatment-related factors (type and dose of chemotherapy, route of administration) and patient-related factors (gender, age, previous history of CINV, alcohol ingestion, anxiety, concurrent use of other therapies) [13]. Some complications of CINV may include loss of appetite, electrolyte imbalance, dehydration, weight loss, prolonged hospitalization, and decreased QoL [11].

Patients are the target of health services, and therefore their opinion and experiences should be addressed in evaluating the effectiveness of anti-emetics regimens and improving health services, especially due to nausea being a subjective symptom that is difficult to describe, making the assessment of CINV severity difficult. In addition to this problem, previous studies showed that there is a gap in physician’s assessment of CINV severity and patient’s experience, leading to a wrong estimation of CINV impact on the patient’s QoL and the possible prescription of ineffective anti-emetics regimen [14]. However, this degree of impact greatly differs from patient to patient, leading to a difference in their response and tolerance to CINV [7].

This inconsistency in understanding the patients’ CINV experience along with the wide variation between patients on how much CINV affected their QoL calls for further studies in different populations. There is a lack of studies on this effect among the Saudi population specifically, and with improving patients’ experience being one of Saudi Arabia’s national transformation initiatives, as well as improving the quality and efficiency of healthcare services [15], more research in this field is necessary. Therefore, the aim of this study was to explore breast cancer patients’ perception of their experience with CINV and how it affects their QoL. A secondary objective is to identify associations between sociodemographic characteristics and CINV impact on QoL.

## Materials and methods

Design

This study was a quantitative retrospective cross-sectional study conducted in Princess Norah Oncology Center (PNOC) in King Abdulaziz Medical City (KAMC) in Jeddah, Saudi Arabia. The study was carried out using a survey.

Setting

KAMC is a tertiary care hospital with around 800 beds. PNOC was established in 2001 and is one of the largest centers for cancer treatment in the area. PNOC includes Adult Medical Oncology, Adult Hematology/Bone Marrow Transplantation (BMT), Pediatric Hematology/Oncology/BMT, Radiation Oncology, Palliative Care, Gynecology Oncology, and Oncology Data & Research Unit. The center has outpatient, inpatient, and radiation therapy services. It accommodates 88 adult oncology inpatient beds and 22 beds for the Bone Marrow Transplant Unit.

Sample of the study

Purposeful sample was selected conveniently. Participants of this study were breast cancer female patients ≥ 18 years of age who had received chemotherapy between January 1, 2018, and July 22, 2019. Exclusion criteria included (a) patients who had other causes of nausea and vomiting such as gastroenteritis and (b) the presence of brain metastasis. The total population at the time of data collection comprised 173 female patients with breast cancer treated at PNOC who fulfilled the inclusion criteria. All of them were invited to participate in this study. A convenient consecutive sampling was used for this study. A total of 98 patients responded.

Data collection

Researchers distributed the survey and collected them from patients who accepted participating in the study at the Oncology Department. Data were collected using two instruments. Several techniques were applied to collect data, including patients record review, structured telephone interviews, and face-to-face interview at the Oncology Clinic. The two instruments are as follows:

1. Sociodemographic characteristics data collected from patients’ files, including age, educational status, marital status, number of pregnancies, work status, and more.

2. A CINV Experience Questionnaire composed of four parts containing 29 items. This questionnaire was compiled by the researchers after extensive literature review. The 29 items were based on two instruments: (a) Functional Living Index-Emesis (FLIE) [16], which is used to evaluate the relationship between emesis and its effects on patient’s QoL, and (b) MASCC Antiemesis Tool (MAT) [17], which is used to evaluate the effectiveness of anti-emetics. The four parts of the questionnaire used are as follows:

a. Items 1 to 4: Experience with nausea and vomiting during the first 24 hours after chemotherapy.

b. Items 5 to 8: Experience with nausea and vomiting during 24 hours to four days after chemotherapy.

c. Items 9 to 22: Rating of how much nausea or vomiting has affected an aspect of QoL during the last chemotherapy cycle.

d. Items 23 to 29: Additional information about chemotherapy treatment, including improvement of CINV with each cycle, side effect of chemotherapy, anti-emesis treatment, other methods to control CINV, and if the information provided about CINV were comprehensive.

The questionnaire was validated by face validity using an expert focus group of 15 members. The group was given a tool containing items regarding clarity, simplicity, appropriateness, relevance, and time needed to complete the questionnaire. Also, an expert breast cancer oncologist opinion was taken on the questionnaire. Some minor modifications were made accordingly before data collection. A pilot study was conducted using a small sample of 15 patients to measure the reliability of the questionnaire using SPSS Version 21 (IBM Corp., Armonk, NY, USA). The instrument’s reliability using Cronbach’s alpha was 0.87. This indicates that the instrument lies in the range of acceptable to good, which means that it is reliable and repeated measures using it can give similar results each time it is used [18]. The instrument was translated into Arabic. To validate the translated tool, back translation was conducted, and expert opinion of an expert in English Linguistics was taken.

Data analysis

Data were managed and analyzed using SPSS Version 21. Descriptive and inferential analyses were performed. Percentage and graphs were used to describe categorical variables, mean with standard deviation was used to describe normally distributed continuous variables, and median with interquartile range was used for non-normally distributed continuous variables. Correlation coefficient, chi-square test, one-way analysis of variance, and two-tailed t-test were used for inferential analysis. Alpha was set at 0.05.

Ethical considerations

Approval of this study was obtained from King Abdullah International Medical Research Centre for the purpose of protection of participants in the study. All participants received an informed consent, with each questionnaire ensuring that the participation is voluntary. This informed consent contained the purpose of the study, research procedure, and a guarantee to maintain anonymity and confidentiality of the information and that no names will be disclosed. The consent stated that participation is voluntary, and participants can withdraw from the study any time. For the telephone structured interview questionnaire, all contents of the informed consent were explained in the presence of a witness during the consent process. The investigators of this study promote the participants’ right based on the ethical principles of respect for human right to full disclosure and principle of justice, which includes the right to fair treatment and the privacy, principle of beneficence and non-maleficence, and, lastly, the principle of autonomy.

## Results

Descriptive statistics

A total of 173 female patients were invited to participate in this study, of whom 98 responded (56.65%) and the rest did not respond due to not answering the telephone, not willing to participate, or due to mortality. The mean age of participants was 51.63 years (SD = 11.50; range: 25-81 years). Of them, 73% were married. Data on sociodemographic characteristics in Table 1 shows that nearly half (49.5%) of them had university or post-graduate education, whereas 18.6% did not receive any formal education. Only 26.8% were working, whereas 11.3% has left their job due to health condition. The mean number of pregnancies was 5.78 ± 2.59.

**Table 1:** Sociodemographic characteristics n = number of subjects

Variable	n	%
Education level		
Not educated	18	18.6
General education	31	32.0
University education	45	46.4
Post-graduate education	3	3.1
Total	97	100.0
Marital status		
Single	8	8.2
Married	72	73.5
Divorced	5	5.1
Widow	13	13.3
Total	98	100.0
Working status		
Unemployed	45	46.4
Working	26	26.8
Left the work due to health	11	11.3
Left the work not due to health	15	15.5
Total	97	100.0
	N	Mean ± SD
Number of pregnancies (times)	90	5.78 ± 2.59
Age (years)	98	51.63 ± 11.50

Frequency table of CINV is shown in Table 2. The median of numbers of acute vomiting episodes was 2.5 (interquartile range = 3; range: 1-12), whereas the median of numbers of delayed vomiting episodes was 2.5 (interquartile range = 4; range: 1-13). The mean nausea severity score was 6.73 ± 2.64 for acute period and 6.34 ± 2.58 for delayed period.

**Table 2:** Frequency of CINV among participants (N = 98) N = number of subjects *Some patients experienced both acute and delayed. CINV, chemotherapy-induced nausea and vomiting

	Chemotherapy-induced nausea, n (%)	Chemotherapy-induced vomiting, n (%)
Occurred	77 (78.6%)	35 (35.7%)
Acute*	51 (52.0%)	15 (15.3%)
Delayed*	65 (66.3%)	26 (26.5%)
Did not occur	21 (21.4%)	63 (64.3%)

Table 3 shows the summary of responses to impact of nausea and vomiting on QoL; the effect of nausea and vomiting on enjoying meals was the most item to have an impact on QoL (80.5% and 71.4% respectively), whereas the effect of imposing hardships on others was the least (37.7% and 45.7% respectively). Most participants showed moderate-to-extreme impact on their QoL due to nausea (74.0%) and vomiting (62.9%).

**Table 3:** Summary of responses to impact on QoL questions QoL, quality of life

Item	% of Total Responses	
	No-to-slight impact	Moderate-to-extreme Impact
Nausea impact on QoL items (N = 77)		
Doing minor household repairs	39.0%	61.0%
Enjoying meals	19.5%	80.5%
Enjoy drinking liquids	28.6%	71.4%
Spending time with family and friends	46.8%	53.2%
Daily functioning	45.5%	54.5%
Personal hardship	41.6%	58.4%
Hardship on others	62.3%	37.7%
Total score	26.0%	74.0%
Vomiting impact on QoL items (N = 35)		
Doing minor household repairs	40.0%	60.0%
Enjoying meals	28.6%	71.4%
Enjoy drinking liquids	40.0%	60.0%
Spending time with family and friends	51.4%	48.6%
Daily functioning	45.7%	54.3%
Personal hardship	45.7%	54.3%
Hardship on others	54.3%	45.7%
Total score	37.1%	62.9%

**Figure 1:**
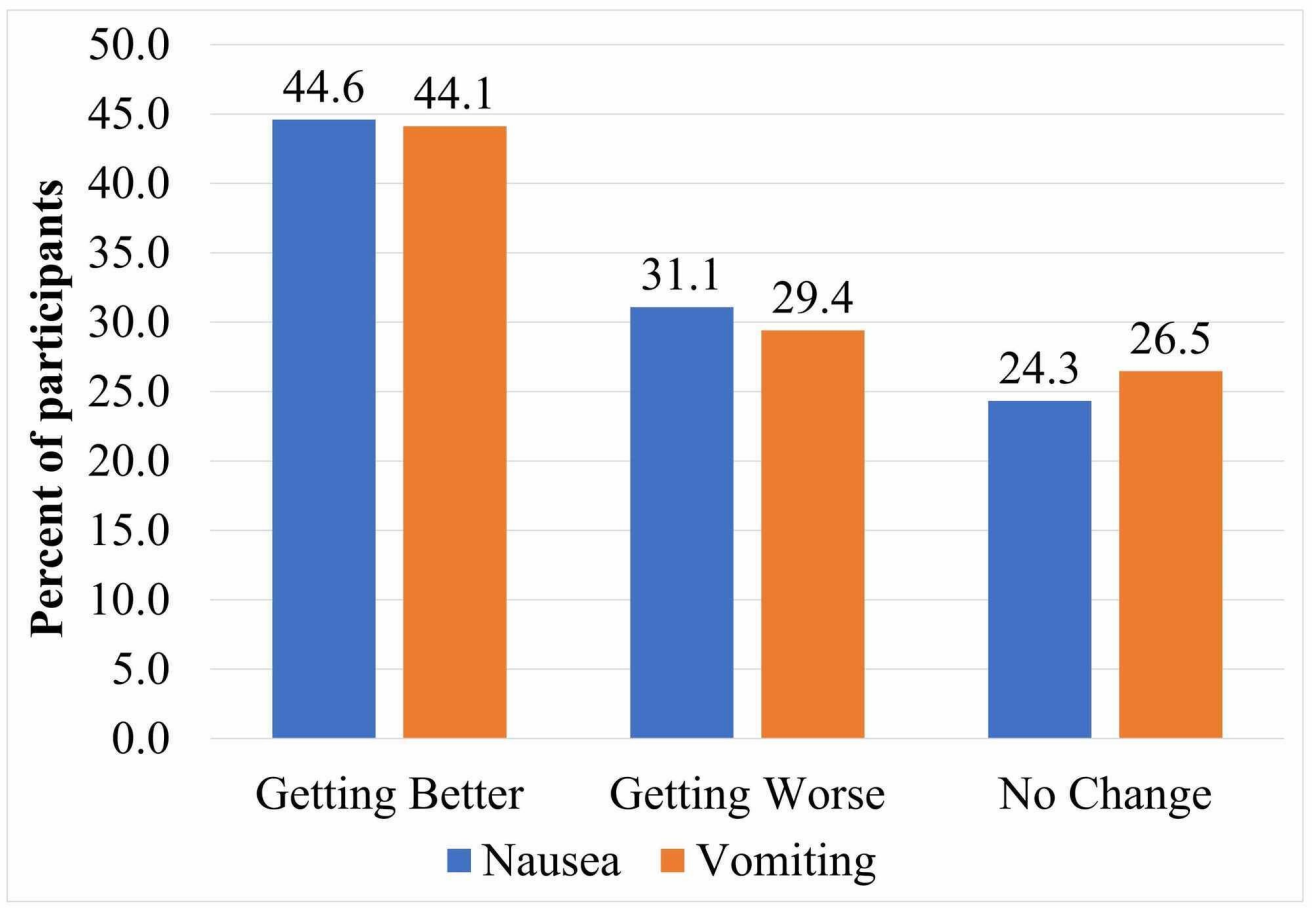
Responses to severity of CINV with each consecutive cycle CINV, chemotherapy-induced nausea and vomiting

Figure 1 shows that almost half of patients felt nausea and vomiting getting better with each consecutive chemotherapy cycle (44.6% and 44.1%, respectively); 57.5% of patients rated anti-emetics as excellent in controlling their CINV symptoms, whereas 22.9% rated them as moderate to good. Overall, 83.5% were completely compliant with their anti-emetics medications as prescribed, and 71.1% has reported that the information provided to them by health care professionals about CINV was completely comprehensive.

**Figure 2:**
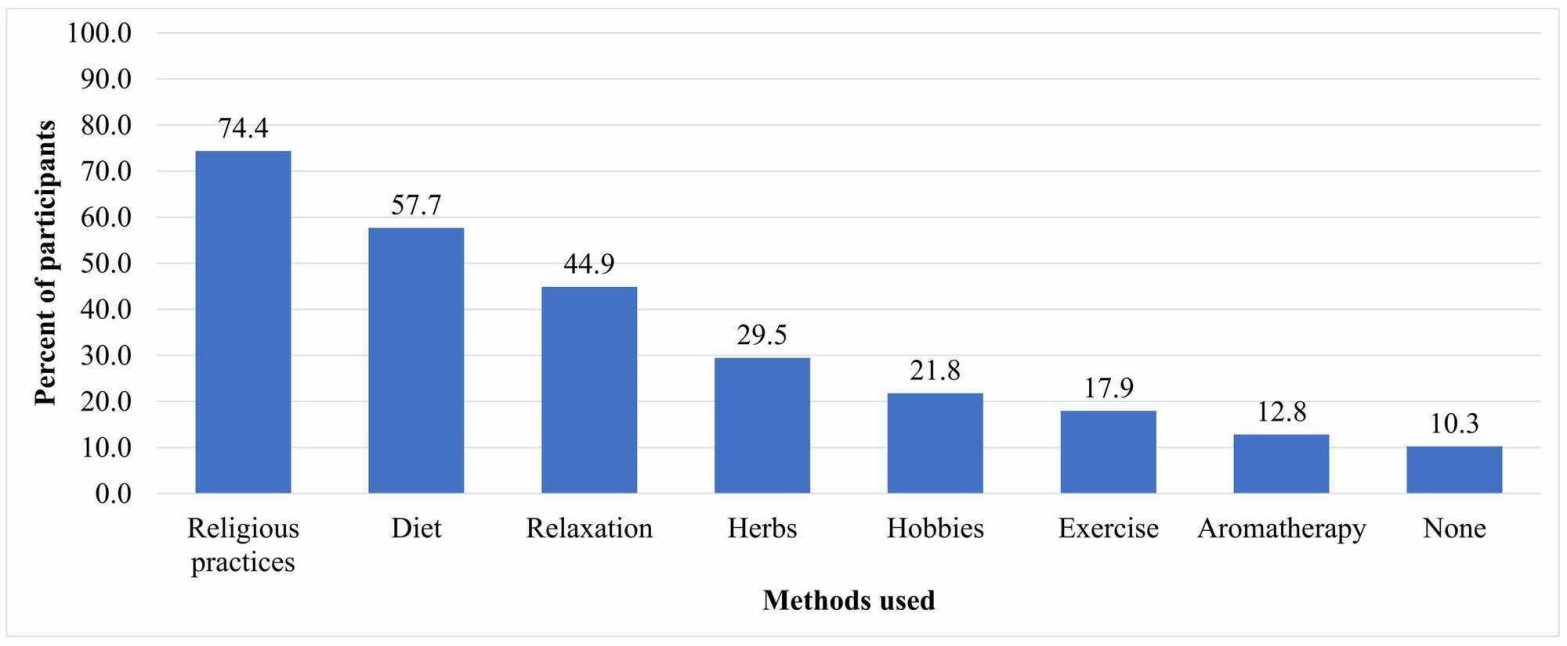
Other methods used to control CINV symptoms CINV, chemotherapy-induced nausea and vomiting

Participants indicated that they used other methods for controlling CINV symptoms (Figure 2). Religious practices were the most common method used (74.4%), whereas aromatherapy was the least common method (12.8%). There were other chemotherapy side effects that accompanied CINV, as reported by patients. These included loss of hair (98.7%), weakness (89.7%), tiredness (88.5%), joints and muscles pain (85.9%), headache (74.4%), diarrhea (61.5%), increased susceptibility for infections (55.1%), numbness (55.1%), and low self-esteem (33.3%).

CINV occurrence and its severity

The results of this study found no significant correlation between sociodemographic characteristics and CINV occurrence and severity (p > 0.05), except for age, which had a weak but significant negative correlation with severity of acute and delayed nausea (p = 0.048; r = -0.278 and p = 0.001, r = -0.391, respectively).

There was a significant negative correlation between delayed nausea severity and patients’ rating on how excellent anti-emetics helped them in controlling CINV symptoms (p = 0.009; r = -0.327). This correlation was not significant with the severity of acute nausea (p = 0.097).

CINV impact on QoL

Participants who had lower delayed nausea severity (mean = 4.93 ± 2.76) were less likely to have a high impact of nausea on their QoL compared to those with higher delayed nausea severity (mean = 6.73 ± 2.417; CI = -3.299 to -0.295; p = 0.02). In contrast, there was no significant correlation between acute nausea severity and impact of nausea on QoL (p = 0.895). There was no significant correlation with any of the sociodemographic characteristics and CINV impact on QoL (p > 0.05).

## Discussion

The result of this study demonstrates that CINV is very common among female adult breast cancer patients. More than 78% had CINV despite being completely compliant with their anti-emetics medications, which they rated as effective in controlling CINV symptoms. This indicates that anti-emetics are still not enough in controlling CINV symptoms. This high prevalence also appears in previous studies, which found that nearly 70-80% reported having CINV [10,19]. Also, the finding in this study that there is a higher incidence of CINV during the delayed period than the acute period supports previous literature [19,20].

Unfortunately, more than two-thirds of participants had a moderate-to-extreme impact of CINV on their QoL. This impact was greatly varied across different aspects, with enjoying meals being the most affected aspect of QoL. But still, the other aspects also showed high impact on QoL, proving that CINV is a very troublesome side effect of chemotherapy. This result is supported by Salihah et al. who found that CINV affected different aspects of life, particularly eating, physical, emotional, and social functions [7]. Another study found that CINV interferes with the QoL of patients, with the impact on eating being the most reported effect [21].

Participants felt that their CINV got better with each consecutive cycle. This result is congruent with that of Hernandez Torres et al., who found that CINV among breast cancer patients gets better with each subsequent cycle [22]. In contrast, other studies showed the opposite, where subsequent cycles showed an increase in CINV severity, although those studies were conducted on CINV patients generally with a wide variation on chemotherapy medications and not just breast cancer patients [23].

Participants generally rated anti-emetics highly in controlling their CINV symptoms regardless of symptoms severity. Salihah et al. indicated that two-thirds of study participants expressed their satisfaction with pharmacological anti-emetics as being useful [7]. Disagreeing with this finding is a study by Molassiotis et al., who found that patients who had severe nausea felt that the medications were not effective, but this study was conducted in 2008, whereas anti-emetics have shown major improvements in the last 10 years [21].

Patients claimed that they are highly compliant with their anti-emetics medications. Although this claim cannot be proved or refuted, a previous study conducted on specific age groups revealed that up to 60% of elderly were actually not compliant with their medications, which is a similar age group to the majority of our study’s participants [24]. Another study reported that 42% of breast cancer patients did not adhere to post-chemotherapy anti-emetics protocols [25].

A previous study conducted by Gozzo et al., reported that not all patients had orientation about CINV, and those who received information were not comprehensive [10]. This problem was not found in this study as all patients indicated that they had received enough information about CINV, with most of them rating the information as completely comprehensive. This education is essential as increased knowledge and training on how chemotherapy patients can control their symptoms has proven to significantly improve QoL [26].

Majority of participants tend to use different non-pharmacological methods to control symptoms of CINV. Since this study was conducted in Saudi Arabia and all participants were Muslims, it is not surprising that an increase in religious practices was the most common method used. Salihah et al. in their qualitative study on Muslim Malay breast cancer patients reported that increase in religious practices resulted in a more positive outlook and better psychological status, hence improving their QoL [7]. In this regard, a systemic review reported that diet and relaxation techniques were likely to be beneficial in controlling CINV, whereas exercise, herbs, and aromatherapy still need further exploration [27]. Incidentally, diet and relaxation techniques were the second and third most common non-pharmacological methods used by patients in this study, respectively.

The negative impact on QoL of breast cancer patients can be due to several other side effects of chemotherapy other than nausea and vomiting and should be considered when interpreting these findings. Studies on chemotherapy patients indicated similar results showing hair loss, weakness, and muscle and joints pain as the most occurring symptoms. But still, patients rated nausea as the worst side effect of chemotherapy [22,28].

Regarding statistical association of sociodemographic characteristic with CINV occurrence and severity of sociodemographic characteristic with QoL, surprisingly there was no significant association or difference except for a weak negative correlation of age with nausea severity but not of age with CINV occurrence itself, although other studies indicated significant association of age with CINV occurrence [24]. This result shows that different sociodemographic characteristics have no significant impact on CINV occurrence or an effect on QoL.

This study had some limitations, such as being conducted in a single center, a relatively short period of 18 months, and a small number of participants. Since the total population was invited to participate and chemotherapy is a dreadful experience, a low response rate and some degree of recall bias are inevitable.

Recommendations

CINV is a common side effect for patients who are receiving chemotherapy. Patients will much be benefited from psychological counselling. CINV can be prevented and treated through multimodal treatment including non-pharmacological therapy options. Treatment strategies be individualized based on each patient’s condition.

Implications

Patient experience in general and specifically of CINV is very important factor that should be considered in informing clinical practice because it affects the patients’ quality of live and is associated with a high-cost burden. Patient experience should play a role as evidence in healthcare decision-making.

## Conclusions

Even with good education about CINV and having highly effective anti-emetics with complete compliance, CINV was still highly prevalent among Saudi adult female breast cancer patients, and their QoL was severely affected. This calls for further research on finding new strategies alongside developing more effective anti-emetics in order to have better control of CINV symptoms and improve patients’ QoL.

Future research studies could include a larger group of participants and could be conducted in other settings. In addition, future research could be done including patients who use chemotherapy to treat other types of cancers for comparison.

## References

[1] (2020). Cancer today - Breast fact sheet. https://gco.iarc.fr/today/data/factsheets/cancers/20-Breast-fact-sheet.pdf..

[2] (2020). Cancer today - Saudi Arabia fact sheet. https://gco.iarc.fr/today/data/factsheets/populations/682-saudi-arabia-fact-sheets.pdf.

[3] (2020). NCCN Clinical Practice Guidelines in Oncology (NCCN Guidelines®) Antiemesis Version 2.2020. https://www.nccn.org/professionals/physician_gls/pdf/antiemesis.pdf.

[4] Roila F, Molassiotis A, Herrstedt J (2016). 2016 MASCC and ESMO guideline update for the prevention of chemotherapy- and radiotherapy-induced nausea and vomiting and of nausea and vomiting in advanced cancer patients. Ann Oncol.

[5] Hesketh PJ, Kris MG, Basch E (2017). Antiemetics: American Society of Clinical Oncology Clinical Practice Guideline Update. J Clin Oncol.

[6] Lotfi-Jam K, Carey M, Jefford M, Schofield P, Charleson C, Aranda S (2008). Nonpharmacologic strategies for managing common chemotherapy adverse effects: a systematic review. J Clin Oncol.

[7] Salihah N, Mazlan N, Lua PL (2016). Chemotherapy-induced nausea and vomiting: exploring patients' subjective experience. J Multidiscip Healthc.

[8] Genç F, Tan M (2015). The effect of acupressure application on chemotherapy-induced nausea, vomiting, and anxiety in patients with breast cancer. Palliat Support Care.

[9] Aapro M, Molassiotis A, Dicato M (2012). The effect of guideline-consistent antiemetic therapy on chemotherapy-induced nausea and vomiting (CINV): the Pan European Emesis Registry (PEER). Ann Oncol.

[10] Gozzo Tde O, de Souza SG, Moysés AM, Panobianco MS, de Almeida AM (2014). [Incidence and management of chemotherapy-induced nausea and vomiting in women with breast cancer]. Rev Gaucha Enferm.

[11] Al Qadire M (2018). Chemotherapy-induced nausea and vomiting incidence and management in Jordan. Clin Nurs Res.

[12] Vidall C (2011). Chemotherapy induced nausea and vomiting: a European perspective. Br J Nurs.

[13] Lavdaniti M, Tsitsis N (2014). Investigation of nausea and vomiting in cancer patients undergoing chemotherapy. Health Psychol Res.

[14] Rha SY, Song SK, Lee CE, Park Y, Lee J. (2016). Gaps exist between patients' experience and clinicians' awareness of symptoms after chemotherapy: CINV and accompanying symptoms. Support Care Cancer.

[15] (2020). National transformation program delivery plan 2018-2020. https://vision2030.gov.sa/sites/default/files/attachments/NTP%20English%20Public%20Document_2810.pdf.

[16] Lindley CM, Hirsch JD, O'Neill CV, Transau MC, Gilbert CS, Osterhaus JT (1992). Quality of life consequences of chemotherapy-induced emesis. Qual Life Res.

[17] Molassiotis A, Coventry PA, Stricker CT (2007). Validation and psychometric assessment of a short clinical scale to measure chemotherapy-induced nausea and vomiting: the MASCC antiemesis tool. J Pain Symptom Manage.

[18] Tavakol M, Dennick R (2011). Making sense of Cronbach's alpha. Int J Med Educ.

[19] Hassan BA, Yusoff ZB (2010). Negative impact of chemotherapy on breast cancer patients QOL - utility of antiemetic treatment guidelines and the role of race. Asian Pac J Cancer Prev.

[20] Booth CM, Clemons M, Dranitsaris G (2007). Chemotherapy-induced nausea and vomiting in breast cancer patients: a prospective observational study. J Support Oncol.

[21] Molassiotis A, Stricker CT, Eaby B, Velders L, Coventry PA (2008). Understanding the concept of chemotherapy-related nausea: the patient experience. Eur J Cancer Care (Engl).

[22] Hernandez Torres C, Mazzarello S, Ng T (2015). Defining optimal control of chemotherapy-induced nausea and vomiting-based on patients' experience. Support Care Cancer.

[23] Dranitsaris G, Molassiotis A, Clemons M (2017). The development of a prediction tool to identify cancer patients at high risk for chemotherapy-induced nausea and vomiting. Ann Oncol.

[24] Jakobsen JN, Herrstedt J (2009). Prevention of chemotherapy-induced nausea and vomiting in elderly cancer patients. Crit Rev Oncol Hematol.

[25] Chan A, Low XH, Yap KY (2012). Assessment of the relationship between adherence with antiemetic drug therapy and control of nausea and vomiting in breast cancer patients receiving anthracycline-based chemotherapy. J Manag Care Pharm.

[26] Bayati M, Molavynejad S, Taheri N, Cheraghian B (2019). Investigating the effect of Integrated Educational Program on the Quality of Life among Cancer Patients: A Clinical Trial Study. Asian Pac J Cancer Prev.

[27] Tipton JM, McDaniel RW, Barbour L, Johnston MP, Kayne M, LeRoy P, Ripple ML (2007). Putting evidence into practice: evidence-based interventions to prevent, manage, and treat chemotherapy-induced nausea and vomiting. Clin J Oncol Nurs.

[28] Sasaki H, Tamura K, Naito Y (2017). Patient perceptions of symptoms and concerns during cancer chemotherapy: 'affects my family' is the most important. Int J Clin Oncol.

